# Supplementary material for: Illustrations of Coping and Mental Well-Being of Adolescents Living with HIV in Cape Town, South Africa During COVID: A Photovoice Study
Source: Int J Environ Res Public Health. 2024 Nov 14;21(11):1517. doi: 10.3390/ijerph21111517 (PMC11593735; doi:10.3390/ijerph21111517)
Supplement: Supplementary file 1 [file ijerph-21-01517-s001.zip › ijerph-3268095-supplementary.pdf]

Supplementary material

Table S1. Summary description of participants (N = 21).

Participant Number	Age (in Years)	Sex
1	15	Female
2	17	Male
3	18	Female
4	17	Female
5	19	Female
6	18	Female
7	18	Female
8	14	Male
9	19	Male
10	18	Female
11	16	Male
12	18	Female
13	18	Female
14	18	Female
15	18	Male
16	17	Male
17	15	Female
18	19	Male
19	16	Female
20	18	Male
21	17	Female
